# Biological Age Estimation From the Age Gap Using Deep Learning Integrating Morbidity and Mortality: Model Development and Validation Study

**DOI:** 10.2196/71592

**Published:** 2025-09-10

**Authors:** Seong-Eun Moon, Ji Won Yoon, Jae Hyun Bae, Shinyoung Joo, Yoo Hyung Kim, Bon Hyang Lee, Seokho Yoon, Haanju Yoo, Young Min Cho

**Affiliations:** 1 NAVER AI Lab, Seongnam, Republic of Korea; 2 Division of Endocrinology and Metabolism, Department of Internal Medicine, Seoul National University Hospital Healthcare System Gangnam Center, Seoul, Republic of Korea; 3 Department of Internal Medicine, Seoul National University College of Medicine, 101 Daehak-ro, Jongno-gu, Seoul, 03080, Republic of Korea, 82 0220721965; 4 Division of Endocrinology and Metabolism, Department of Internal Medicine, Seoul National University Hospital, Seoul, Republic of Korea; 5 DaNaA Data, Seoul, Republic of Korea

**Keywords:** aging, deep learning, health status, morbidity, mortality, risk assessment

## Abstract

**Background:**

Biological age (BA) is increasingly recognized as a valuable alternative to chronological age (CA) for assessing an individual’s health and aging status. However, existing models are based on limited clinical parameters and have not thoroughly integrated morbidity and mortality data.

**Objective:**

This study aimed to develop and validate a novel transformer-based model, referred to as the BA – CA gap model, for BA estimation that incorporates morbidity and mortality information to improve predictive accuracy and enhance clinical use in the early identification of the risk of age-related diseases.

**Methods:**

We retrospectively analyzed data from 151,281 adults aged 18 years or older who underwent routine health checkups between 2003 and 2020. Participants were classified into normal, predisease, and disease groups based on comorbidities (diabetes mellitus, hypertension, and dyslipidemia) to evaluate the model’s ability to discriminate health status along a clinically relevant spectrum. Variables with less than 50% missingness had missing values imputed using the mean, while features with 50% or more missingness were excluded. We develop a custom transformer architecture that learns multiple objectives simultaneously, including input feature reconstruction, BA and CA alignment, health status discrimination, and mortality prediction. Model training used unsupervised and self-supervised strategies. We compared our model’s performance with conventional BA estimation approaches, including Klemera and Doubal’s method, a CA cluster-based model, and a deep neural network, by examining BA gap distributions, health status stratification, and mortality prediction.

**Results:**

The proposed BA – CA gap model provided a more accurate reflection of health status and superior stratification of mortality risk than existing methods. The model effectively distinguished among normal, predisease, and disease groups, with a clear gradient of BA gap values. Kaplan-Meier analyses demonstrated stronger discrimination of future mortality in men, while a similar but not statistically significant trend was observed in women. Sensitivity analyses across multiple random splits and training subsets confirmed the robustness of the model’s performance.

**Conclusions:**

By integrating morbidity and mortality information within a transformer-based framework, the BA – CA gap model offers a more granular and clinically meaningful assessment of aging and health status than CA alone. This approach supports the potential for personalized health management and risk stratification, although external validation in diverse populations is warranted to further confirm its generalizability.

## Introduction

Biological aging is a critical determinant of functional decline, age-related diseases, and mortality, distinct from chronological aging, which simply measures time since birth [1,2]. Biological age (BA) is shaped by a complex interplay of genetics, environmental exposures, modifiable lifestyle factors, and chronic diseases, making it highly individualized and multifaceted [3,4]. Distinguishing BA from chronological age (CA) has important implications for preventive health care [5], risk stratification for frailty [6], and extending healthy lifespan [7].

Over recent decades, numerous methods have been developed to estimate BA, ranging from composite indices of clinical markers [6] to advanced models using omics data [8] and machine learning techniques [9]. Although these methods have proven valuable in aging research, they face notable challenges. Traditional models frequently use CA as the principal anchor, assuming a close alignment between BA and CA, which limits their applicability across diverse populations and health statuses [10]. Additionally, many models are trained exclusively on healthy or homogeneous cohorts, raising concerns about their generalizability to individuals with multimorbidity or differing baseline risks [11].

Recent advances in artificial intelligence (AI), such as deep learning, offer significant promise for modeling the nonlinear and high-dimensional relationships inherent in biological aging [12]. While these approaches have shown potential for assessing age-related outcomes using clinical data [13], many published BA estimation models do not explicitly incorporate morbidity or mortality data during model training [14]. As a result, their relevance to clinical practice and their ability to predict meaningful health outcomes may be limited. Moreover, robust frameworks that integrate longitudinal health events across heterogeneous populations are still lacking [15], further hampering translation into real-world practice.

This study addresses these gaps by proposing a transformer-based deep learning model for BA estimation that integrates both morbidity and mortality data from routine clinical practice. Unlike conventional approaches, our model is designed to jointly learn the reconstruction of clinical features, discrimination of health status, prediction of all-cause mortality, and semantic alignment of BA and CA. This yields a single, interpretable BA – CA gap metric that reliably reflects both biological aging and prospective health risk.

We hypothesize that by leveraging morbidity and mortality data within a transformer-based deep learning framework, the model will provide BA estimates that more accurately reflect individual health status and better predict adverse outcomes than conventional approaches. To enhance accuracy and generalizability, our model was trained and validated using a large-scale, longitudinal health checkup dataset comprising more than 150,000 adults. We compared its performance with established BA estimation methods, focusing on the discrimination of morbidity status and prediction of future mortality.

## Methods

### Study Cohort

The study cohort included individuals who underwent health checkups at the Seoul National University Hospital Healthcare System Gangnam Center between 2003 and 2020. The dataset comprised initial visit records of 151,281 individuals who voluntarily participated in routine health examinations. Participants underwent these checkups, either as part of or in addition to examinations mandated by the Korean National Health Insurance Service, a single-payer system with near-universal coverage [16].

### Ethical Considerations

The study protocol was approved by the Institutional Review Board (IRB) of Seoul National University Hospital (IRB number 2104-098-1211) and was conducted in accordance with the Declaration of Helsinki and applicable regulations [17]. All data were retrospectively collected and fully anonymized by the institution’s data management team using a secure coding process that removed direct identifiers (eg, names and resident registration numbers), thus ensuring participant confidentiality. As the analysis involved only deidentified data used for secondary research purposes, the IRB waived the requirement for informed consent, consistent with institutional guidelines. This study follows the STROBE (Strengthening the Reporting of Observational Studies in Epidemiology) guidelines [18].

### Measurements

Comorbidities, including diabetes mellitus (DM), hypertension, dyslipidemia, cardiovascular disease, and cancer, were assessed through standardized questionnaires. Information regarding medication use for DM, hypertension, and dyslipidemia was also collected. Additional data were obtained from anthropometric measurements, blood tests, bioelectrical impedance analysis, and pulmonary function tests. Mortality data, linked to Statistics Korea, were analyzed from January 2004 to December 2020. Deaths due to external causes (*International Classification of Diseases, Tenth Revision* [*ICD-10*] codes: S00-S99, T00-T98, U01-U03, V01-V99, W00-W99, X00-X84, X85-Y05, Y08-Y09, Y10-Y36, and Y85-Y89) were excluded.

### Data Preprocessing

Features with ≥50% missing data were excluded. Remaining missing values were imputed using the mean of observed values, recognizing that this approach could reduce variability and introduce bias, but were chosen for pragmatic management of a large, complex dataset.

### Population Classification for BA Estimation

Participants were categorized into 3 health status groups: normal, predisease, and disease. Normal status was defined as having fasting glucose <100 mg/dL and glycated hemoglobin (HbA_1c_) <5.7%, systolic blood pressure (SBP) <120 mm Hg and diastolic blood pressure (DBP) <80 mm Hg, low-density lipoprotein cholesterol (LDL-C) <100 mg/dL, triglycerides <150 mg/dL, and high-density lipoprotein cholesterol (HDL-C) ≥60 mg/dL. Predisease status included fasting glucose 100‐125 mg/dL or HbA_1c_ 5.7%‐6.4% (ie, prediabetes), SBP 120‐139 mm Hg or DBP 80‐89 mm Hg (ie, elevated blood pressure), LDL-C 100‐159 mg/dL, triglycerides 150‐199 mg/dL, or HDL-C 40‐59 mg/dL (ie, borderline lipid levels). Disease status was defined as having fasting glucose ≥126 mg/dL, HbA_1c_≥6.5%, or the use of antidiabetic medications (ie, DM); SBP ≥140 mm Hg, DBP ≥90 mm Hg, or the use of antihypertensive medications (ie, hypertension); or LDL-C ≥160 mg/dL, triglycerides ≥200 mg/dL, HDL-C <40 mg/dL, or the use of lipid-lowering medications (ie, dyslipidemia). Missing data that had been imputed using mean values were excluded from the categorization process. Consequently, individuals with missing values for the relevant variables were classified into the normal category.

The population was further classified into four groups based on morbidity: (1) normal population, (2) normal and predisease population, (3) entire population, and (4) entire population excluding outliers (individuals within mean, 2 SDs of key variables, including HbA_1c_, SBP, DBP, LDL-C, triglycerides, HDL-C, and waist circumference).

### Selection of Feature Sets

We defined 7 health-related domains (anemia, adiposity, inflammation, kidney function, lung function, metabolism, and nutrition), each linked to a representative clinical phenotype. Feature selection was guided by expert opinion (JYW, JHB, YHK, and YMC) and correlation with CA. The relationships between features and CA were analyzed using 3 methods: Pearson correlation coefficient (PCC) for linear relationships, Spearman rank-order correlation coefficient (SROCC) for monotonic relationships, and mutual information (MI) for informational dependence. Analyses were performed for both the entire and normal populations. Features in the top 10% of PCC, SROCC, and MI relative to CA are shown in Table S1 in Multimedia Appendix 1. Features with >50% missing data were excluded; remaining missing values were imputed as described in the “Data Preprocessing” section.

Three feature sets were developed: a base set (13 features), a morbidity-related set (the base set plus fasting glucose, SBP, DBP, LDL-C, triglyceride, and HDL-C), and the entire set (88 features; Table S2 in Multimedia Appendix 1). The number of features used in modeling varied according to data division, with CA-derived features excluded. Table S3 in Multimedia Appendix 1 summarizes the proportion of missing data for key features. Despite the capability of transformer architectures to process high-dimensional data, preliminary feature selection was conducted to reduce dimensionality, minimize noise, and promote stable convergence, thereby enhancing computational feasibility and interpretability.

### Modeling for BA Prediction

**Figure 1.**
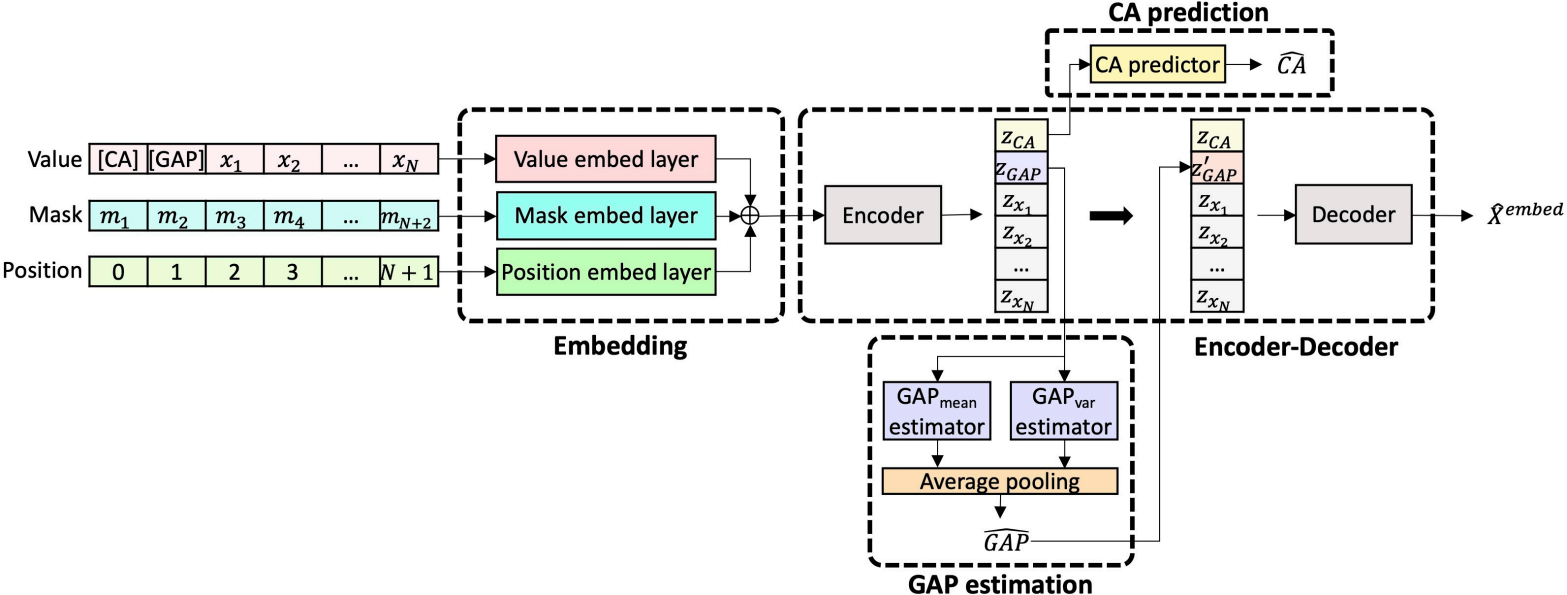
Overview of the model architecture of gap-based biological age estimation. The model predicts biological age (BA) from clinical feature sets using a unified encoder-decoder framework. Input data are embedded with special tokens for chronological age (CA) and the BA – CA gap, along with mask and positional encodings to account for missing values and input structure. The encoder generates latent feature representations, which are used for both CA prediction and gap estimation. The decoder reconstructs the embedded input. The model is trained via a multitask loss that integrates CA prediction, contrastive learning for morbidity, maximizing correlation with mortality, and enforcing semantic consistency of the BA – CA gap. Separate models are developed for men and women to account for sex differences.

The model predicts BA by using 3 feature sets, simultaneously training for encoder-decoder reconstruction, CA prediction, and gap estimation. The model comprises 4 key components: CA prediction, contrastive learning for morbidity, maximizing correlation with mortality, and gap semantic consistency (Figure 1).

#### Embedding

Each clinical feature or questionnaire response was standardized and mapped to an embedding vector via a fully connected layer. Input data (x) sequences included feature embeddings and 2 special tokens: [CA] (chronological age) and [GAP] (the BA gap to be estimated). CA was normalized and embedded as the [CA] token; [GAP] was initially a placeholder, updated during inference to predict the BA gap. Missing values were marked using an imputation mask (m), with 0 indicating original values and 1 indicating imputed values. The input embedding is formulated as:


$$X^{embed}=e_{f}\left(x\right)+e_{m}\left(m\right)+e_{p}\left(p\right)$$

where $e_{f}$ is the feature embedding layer, $e_{m}$ the masking embedding, and $e_{p}$ the positional embedding.

#### Encoder-Decoder

The Performer, a Transformer with linear self-attention, was used as the encoder-decoder. The encoder generated latent features for the [CA] and [GAP] tokens, which the decoder used to reconstruct the embedded input. Reconstruction loss ($L_{recon}$) for K samples is calculated using the mean squared error:


$$L_{recon}=\frac{1}{K\left(N+2\right)}\sum_{i=1}^{K}\sum_{j=1}^{N+2}{\left({\hat{X}}_{i,j}^{embed}-X_{i,j}^{embed}\right)}^{2}$$

where $X_{i,j}^{embed}$ represents the embedded description of the j-th feature of the i*-*th sample.

#### CA Prediction

CA was predicted from the latent [CA] token, processed through 2 fully connected layers. The loss combined mean squared error and *R*^2^ to improve accuracy and encourage CA-relevant encoding. Given the predicted *ŷ* and true CA *y*, the training loss $L_{CA}$ is:


$$L_{\text{CA}}=\frac{1}{K}\sum_{i=1}^{K}\left(({\hat{y}}_{i}-y_{i}{)}^{2}+\lambda_{{\text{CA}}_{R2}}\left(1-\frac{\sum_{i=1}^{K}({\hat{y}}_{i}-y_{i}{)}^{2}}{\sum_{i=1}^{K}(y_{i}-\bar{y}{)}^{2}}\right)\right)$$

#### Gap Estimation

Rather than directly predicting BA, the model estimated the gap between BA and CA using the [GAP] token, modeled as a probability distribution and refined through multiple loss components. A negative BA – CA gap indicates slower biological aging, while a positive gap reflects accelerated aging.

 1. Distribution alignment loss ($L_{dist}$) used the maximum mean discrepancy to align the latent features ($Z_{GAP}$) with a normal distribution $N\left(0,1\right)$:


$$L_{dist}=MMD\left(p\left(g|Z_{GAP}\right),\;N\left(0,1\right)\right)$$

2. Consistency loss ($L_{consist}$) ensured independence between the latent [CA] and [GAP] tokens:

$$L_{consist}=\frac{1}{K}\sum_{i=1}^{K}{\left(f_{GAP_{mean}}\left(Z_{GAP}\right)-f_{GAP_{mean}}\left(Z_{GAP}^{*}\right)\right)}^{2}$$

where $f_{GAP_{mean}}$ is the gap mean inference network, and $Z_{GAP}^{*}$ is a shuffled or alternative version for independence.

  3. Contrastive loss for morbidity ($L_{contrast}$) contrasted $g_{org}$ (original gap) and $g_{corr}$ (corrected gap) estimates:


$$L_{\text{contrast}}=\max\left(0,\frac{1}{K}\sum_{i=1}^{K}\left(\gamma -g_{org}-g_{corr}\right)\right)$$

with γ as a tunable margin parameter.

  4. Mortality loss ($L_{mort}$) enforced negative correlation between the predicted gap and observed time-to-death using PCC:


$$L_{mort}=1+PCC_{g,time\_to\_death}$$

The total loss (L) was a weighted sum of these components:


$$L=\lambda_{1}L_{recon}+\lambda_{2}L_{CA}+\lambda_{3}L_{dist}+\lambda_{4}L_{consist}+\lambda_{5}L_{contrast}+\lambda_{6}L_{mort}$$

where $L_{recon}$ is the reconstruction loss, $L_{CA}$ is the loss for CA prediction, and $\lambda_{i}$ are hyperparameters controlling each loss term. The loss weight values were set to $\lambda_{1}=1$, $\lambda_{2}=0.01$, $\lambda_{3}=0.001$, $\lambda_{4}=1$, $\lambda_{5}=10$, and $\lambda_{6}=1$ for the experiments based on model performance on the subset for validation.

### Model Implementation and Training

To account for sex differences, separate models were developed for women and men using PyTorch. Input data were embedded into 128-dimensional vectors. The Performer had 3 layers, 4 attention heads, and 1024 hidden units per feed-forward layer [19]. The model was trained with the AdamP optimizer [20], batch size of 2000, and learning rate of 1e−3, for up to 1000 epochs, with early stopping if validation performance did not improve for 100 successive epochs. For comparison, baseline models were reimplemented following publication of Bae et al [21], using their specified hyperparameters. Features for the Klemera and Doubal’s method (KDM) [22] were selected for original methodology, as alternative feature sets yielded less stable results in preliminary analyses.

The full dataset was randomly split into training (70%), validation (15%), and test (15%) subsets, repeated 3 times with different seeds to create distinct splits. For each split, separate models were trained, and performance metrics were averaged for robustness. Mortality analyses were conducted on test sets, with 956 total samples available for survival analysis.

### Morbidity Discrimination Through 5-Year Averaged BA and CA Gaps

The average gaps between BA and CA were calculated over 5-year intervals to demonstrate improved disease discrimination by the proposed model, compared with existing models, which often yielded implausibly variable BAs (eg, <−10 or >10).

### Statistics and Reproducibility

Continuous variables were reported as mean (SD), and categorical variables as counts and percentages. Student *t* tests compared 2 independent groups. PCC was used to assess linear relationships with CA, SROCC to assess monotonic relationships with CA, and MI to assess informational dependence on CA. Univariate and multivariate linear regressions for time-to-death were conducted using CA, estimated BA, or predicted gap as independent variables. Model performance was benchmarked using KDM [22], CA cluster [14], and deep neural network (DNN) [21] models. Associations with time-to-death were assessed through regression slope, *R*^2^, and PCC. For mortality prediction, participants were classified as healthy and unhealthy groups by gap values, and survival was analyzed with Kaplan-Meier curves and log-rank tests. All reported *P* values were 2-sided, with *P*<.05 considered statistically significant. Analyses were performed using SciPy.

## Results

### Study Participants

We analyzed health checkup data from 151,281 individuals (n=78,847, 52.1% men; n=72,434, 47.9% women), with mean ages of 41.4 (SD 12.1) years for men and 45.7 (SD 12.5) years for women. Participants were categorized at baseline as normal, predisease, and disease (Table 1). Predisease conditions (prediabetes, elevated blood pressure, or borderline lipid levels) were present in 32.4% (n=21,368) of men and 53.4% (n=30,274) of women, while overt diseases (DM, hypertension, or dyslipidemia) were observed in 66.8% (n=56,979) of men and 39.3% (n=38,002) of women. The cohort was classified into 4 morbidity-based groups: normal population, normal and predisease population, entire population, and entire population excluding outliers.

**Table 1.** Baseline characteristics of study participants^a^.

Characteristics	Men	Women
Participants, n (%)	78,847 (100)	72,434 (100)
Age (years), mean (SD)	41.4 (12.1)	45.7 (12.5)
BMI, kg/m^2^		
Data, n (%)	76,431 (96.9)	70,762 (97.7)
Mean (SD)	24.6 (11.3)	21.9 (3.3)
Glycated hemoglobin, %		
Data, n (%)	73,470 (93.2)	68,649 (94.8)
Mean (SD)	5.7 (0.7)	5.6 (0.6)
Morbidity status, n (%)		
Normal	500 (0.8)	4,158 (7.3)
Predisease^b^	21,368 (32.4)	30,274 (53.4)
Disease^c^	56,979 (66.8)	38,002 (39.3)
Glycemic status, n (%)		
Normal	30,997 (40.1)	38,818 (54.4)
Prediabetes	37,806 (49.0)	28,996 (40.6)
Diabetes mellitus	8394 (10.9)	3608 (5.0)
Blood pressure, n (%)		
Normal	28,079 (36.6)	46,068 (64.8)
Elevated blood pressure	26,529 (34.5)	14,615 (20.6)
Hypertension	22,167 (28.9)	10,380 (14.6)
Lipids, n (%)		
Normal	1781 (2.3)	7348 (10.3)
Borderline lipid levels	47,039 (61.2)	52,384 (73.7)
Dyslipidemia	27,994 (36.5)	11,407 (16.0)
Mortality		
Number of events, n (%)	2240 (2.8)	1106 (1.5)
Time-to-death (days), mean (SD)	2794 (1696)	2665 (1689)

a Data are presented as mean (SD) or n (%).

b Predisease is defined as prediabetes, elevated blood pressure, or borderline lipid levels.

c Disease is defined as diabetes mellitus, hypertension, or dyslipidemia.

### Discrimination of Health Status Using Gap Values

Our proposed model and established comparators, including the KDM, CA cluster, and DNN models, were trained to discriminate health status based on the gap between BA and CA. Negative gap values (BA−CA<0) indicate better health, while positive gap values (BA−CA>0) indicate poorer health.

**Figure 2.**
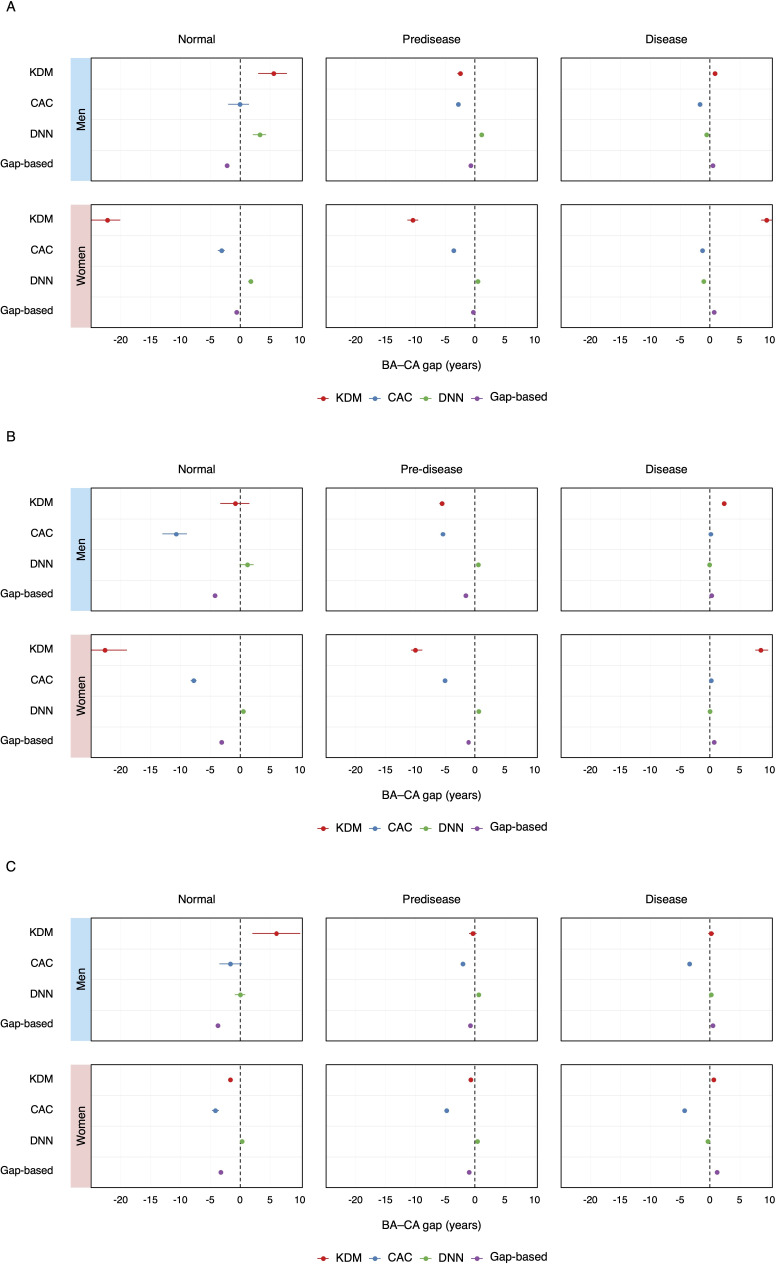
Discrimination of morbidity status by the gap-based model and conventional approaches. Panels show the mean gap between BA and CA with 95% CIs for normal, predisease, and disease groups, using 4 models: KDM (red), CAC (blue), DNN, and the gap-based model (purple). Each panel represents a different feature set: (A) base, (B) morbidity-related, and (C) entire feature set. Within each panel, results are shown separately for men (upper rows) and women (lower rows). The vertical dashed line at zero represents the equivalence of BA and CA. The gap-based model consistently shows negative BA – CA gaps for normal and positive gaps for disease groups, reflecting a clear separation of morbidity status across both sexes and all feature sets. BA: biological age; CA: chronological age; CAC: chronological age cluster; DNN: deep neural network; KDM: Klemera and Doubal’s method.

**Figure 3.**
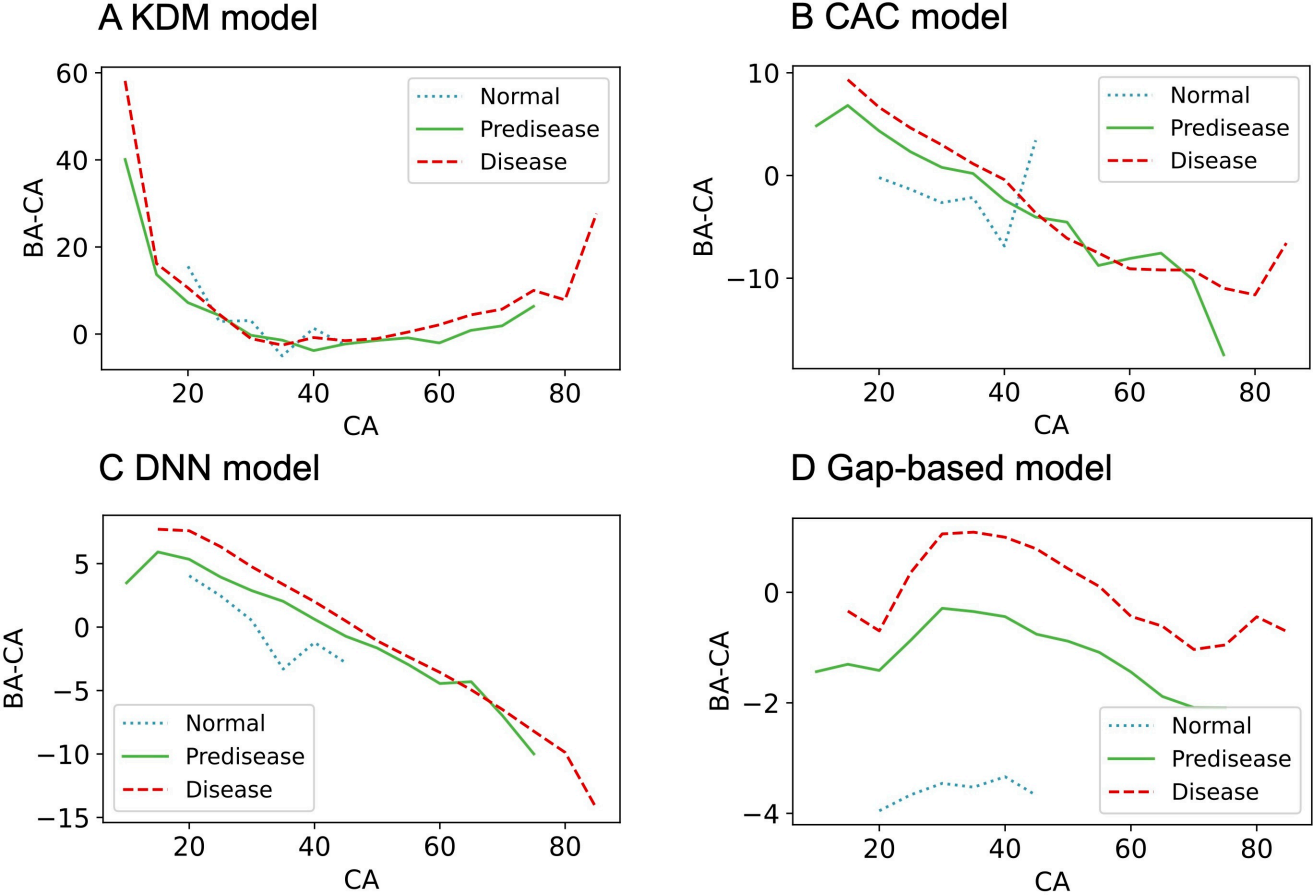
Averaged gaps between BA and CA by health status in men. The mean BA − CA gap is plotted across 5-year CA intervals from normal (dotted line), predisease (solid green), and disease (dashed red) groups. Each panel shows results from a different model: (A) KDM, (B) CAC, (C) DNN, and (D) gap-based model. All models were trained on the entire population using the entire feature set. The gap-based model demonstrates consistent and clinically interpretable separation among health status groups, with normal individuals showing persistently more negative BA – CA gaps across all ages and disease groups showing higher values. BA: biological age; CA: chronological age; CAC: chronological age cluster; DNN: deep neural network; KDM: Klemera and Doubal’s method.

BA estimates were consistently higher in all groups when algorithms were trained on the normal population, resulting in positively skewed gap values (Table S4 in Multimedia Appendix 1). In contrast, models trained on other populations showed negative gaps in the normal group and positive gaps in the disease group, regardless of feature set (Tables S5 and S6 in Multimedia Appendix 1). When trained using the entire feature set on the entire population, the model exhibited the most reasonable trends: negative BA – CA gaps for normal individuals and positive gaps for disease groups, with graded distributions across health status categories (Figure 2A-C and Table S7 in Multimedia Appendix 1). The proposed model outperformed existing models, particularly among men (Figures3A-D  4A-D). The gap between BA and CA remained distinct across different health statuses, even in in-distribution test data (Figure 5A and B).

**Figure 4.**
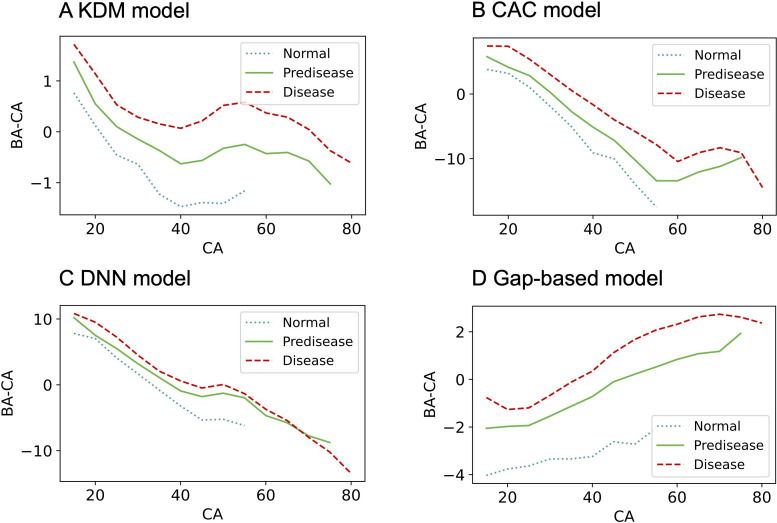
Averaged gaps between BA and CA by health status in women. The mean BA – CA gap is plotted across 5-year CA intervals from normal (dotted line), predisease (solid green), and disease (dashed red) groups. Each panel shows results from a different model: (A) KDM, (B) CAC, (C) DNN, and (D) gap-based model. All models were trained on the entire population using the entire feature set. Among the models, the gap-based model most clearly separates health status groups across age intervals. BA: biological age; CA: chronological age; CAC: chronological age cluster; DNN: deep neural network; KDM: Klemera and Doubal’s method.

**Figure 5.**
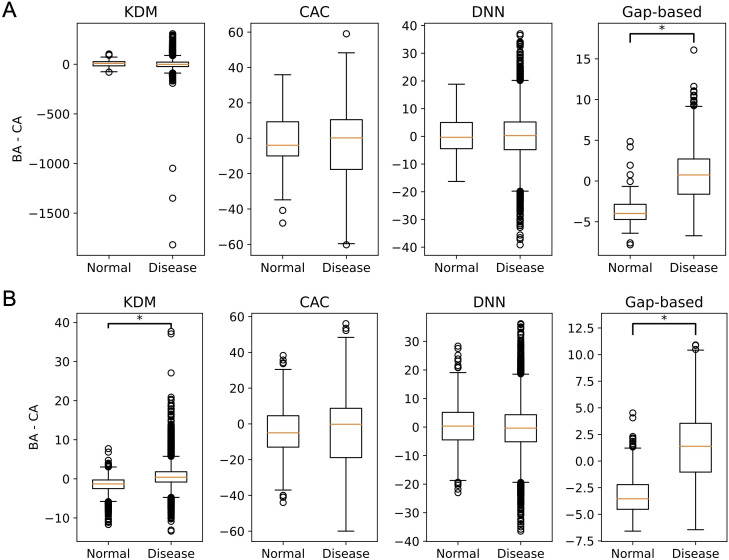
Distributions of the gap between BA and CA in normal and disease groups. Box-and-whisker plots show the distribution of gaps between BA and CA across 4 models: KDM, CAC, DNN, and gap-based model. (A) Results for men. (B) Results for women. All models were trained on the entire population using the entire feature set. For each model, the box indicates the IQR, the line inside the box denotes the median, and whiskers represent 1.5 times the IQR. Outliers are shown as circles. Significant differences between groups (^*^P<.001) are indicated. Among the models, the gap-based model demonstrates the most pronounced separation between normal and disease groups, consistently showing lower BA – CA gaps for normal individuals and higher values for those with disease in both men and women. BA: biological age; CA: chronological age; CAC: chronological age cluster; DNN: deep neural network; KDM: Klemera and Doubal’s method.

Figures3  4 demonstrate that normal status is most common among individuals aged 20‐40 years. This reflects the cohort’s characteristics, as most older participants presented with at least 1 borderline or overt chronic condition, while younger participants (younger than 20 years) often had incomplete data or laboratory abnormalities classifying them as a predisease group. Thus, the observed distribution results from strict health criteria and underlying population characteristics, not from a lack of health individuals outside this age range.

The BA – CA gap robustly reflected the burden of major chronic diseases across multiple domains. Individuals with prediabetes or diabetes exhibited higher gaps than those with normal glycemic status, indicating that even early glucose dysregulation is associated with accelerated biological aging (Tables S8 in Multimedia Appendix 1). Similarly, BA – CA gaps shifted from negative to positive values as blood pressure or lipid levels progressed from normal to overt disease (Tables S9 and S10 in Multimedia Appendix 1). The presence of cardiovascular disease or cancer was also associated with significantly increased BA – CA gaps (Tables S11 and S12 in Multimedia Appendix 1). These patterns were consistent in both women and men. Collectively, the model’s gap serves as a sensitive and clinically relevant marker, integrating the cumulative impact of metabolic risk factors and chronic disease burden on aging.

### Mortality Prediction Using Gap Values

**Figure 6.**
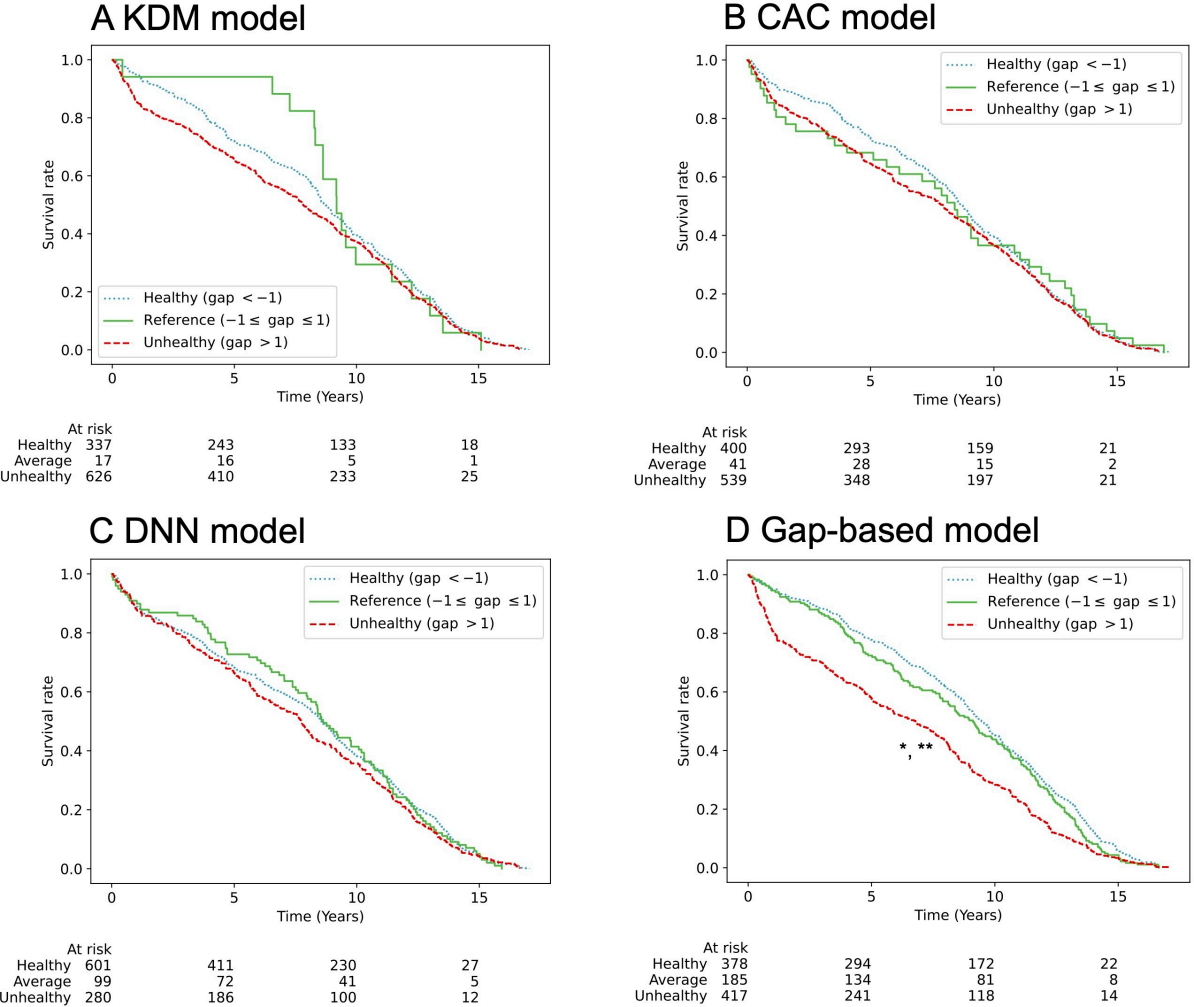
All-cause mortality according to groups by the gap between BA and CA in men. Kaplan-Meier survival curves show all-cause mortality according to the gap between BA and CA in men with death events, using 4 models: (A) KDM, (B) CAC, (C) DNN, and (D) gap-based model. Participants were classified as healthy (gap < −1, dotted blue), unhealthy (gap > 1, dashed red), or reference (–1 ≤ gap ≤ 1, solid green). The number at risk in each group is indicated below each plot. In the gap-based model, both the unhealthy and reference groups show significantly higher mortality risk than the healthy group (*P<.001 vs healthy; **P=.001 vs reference; log-rank test). BA: biological age; CA: chronological age; CAC: chronological age cluster; DNN: deep neural network; KDM: Klemera and Doubal’s method.

**Figure 7.**
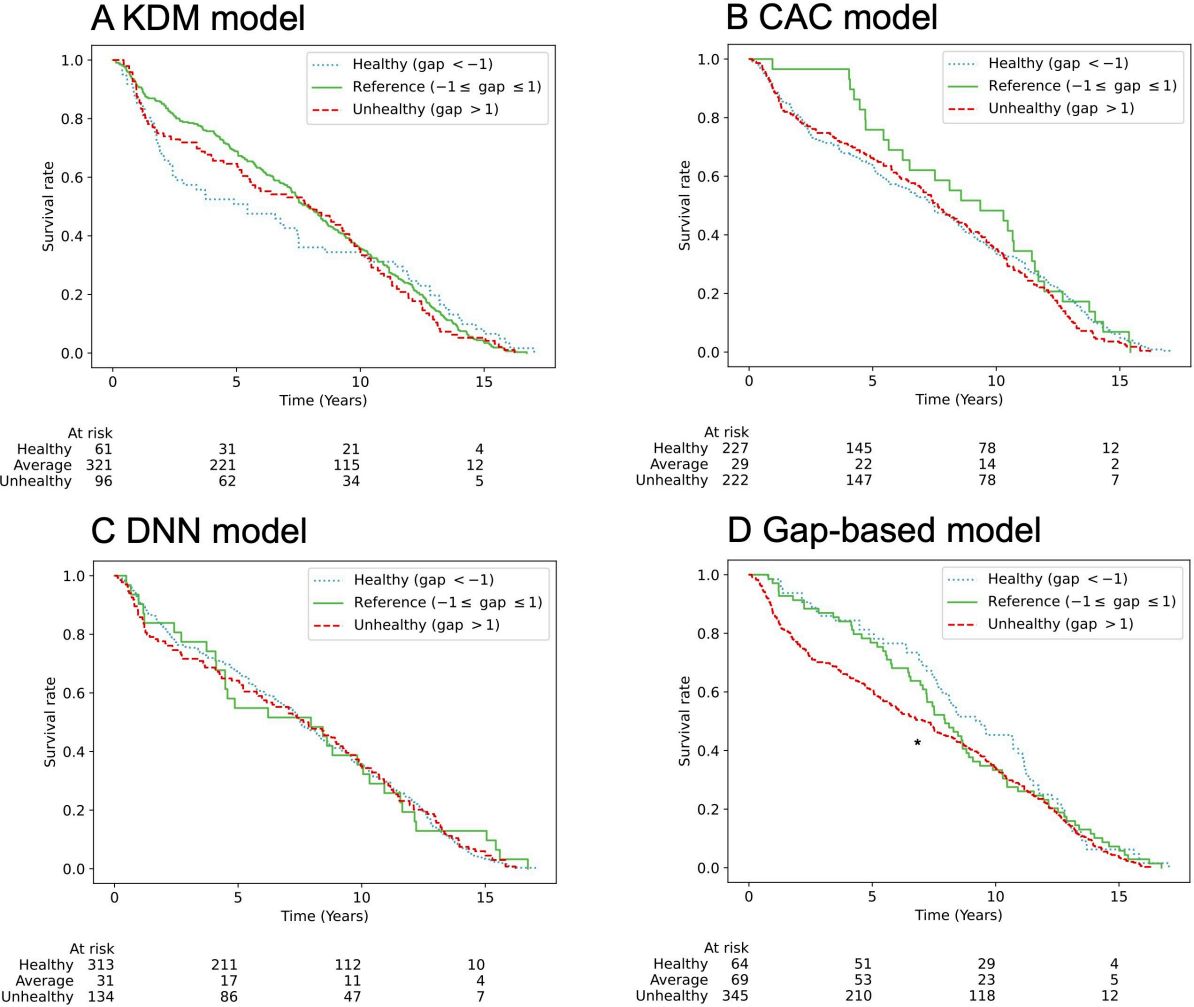
All-cause mortality according to groups by the gap between BA and CA in women. Kaplan-Meier survival curves show all-cause mortality according to the gap between BA and CA in women with death events, using 4 models: (A) KDM, (B) CAC, (C) DNN, and (D) gap-based model. Participants were classified as healthy (gap < −1, dotted blue), unhealthy (gap > 1, dashed red), or reference (–1 ≤ gap ≤ 1, solid green). The number at risk in each group is indicated below each plot. In the gap-based model, the unhealthy group showed a trend toward higher mortality risk than the healthy group (*P=.07 vs healthy; log-rank test). BA: biological age; CA: chronological age; CAC: chronological age cluster; DNN: deep neural network; KDM: Klemera and Doubal’s method.

We evaluated the predictive value of the BA – CA gap for all-cause mortality using time-to-death data. Linear regression analyses demonstrated that the gap-based approach provided more accurate mortality predictions than the BA metric alone (Table S13 in Multimedia Appendix 1). Mortality analyses were performed on the test sets from 3 independent data splits, totaling 956 samples (334, 306, and 316 for each split). Participants were categorized as healthy (gap <−1), unhealthy (gap>1), or reference group (gap between −1 and 1). The proposed model outperformed all existing models in mortality prediction (Table S13 in Multimedia Appendix 1). Kaplan-Meier survival curves confirmed the model’s effectiveness in distinguishing healthy, unhealthy, and reference groups among men (Figure 6A-D). Although survival curve differences did not reach statistical significance among women, trends were consistent: unhealthy individuals exhibited decreased survival, while healthy individuals showed an upward survival trajectory (Figure 7A-D). In contrast, existing models did not effectively discriminate survival outcomes by health status in both men and women.

## Discussion

### Principal Findings

This study introduces a novel deep learning framework for estimating BA by integrating morbidity and mortality information with comprehensive health checkup data. Our transformer-based, multiobjective model, which calculates the gap between BA and CA, outperformed existing BA prediction methods in discriminating disease status and predicting mortality risk. Notably, the BA – CA gap derived from our model showed a stronger correlation with time-to-death than conventional BA estimates, underscoring its potential clinical relevance.

BA estimating methods that heavily rely on CA limit their precision, particularly in populations with heterogeneous health profiles [2]. In contrast, our approach leverages the BA – CA gap and a diverse array of clinical features, resulting in a more individualized assessment of biological aging. Existing DNN models have struggled to accurately reflect morbidity, highlighting the advantages of our gap-based, transformer architecture, which jointly optimizes morbidity classification, mortality prediction, and feature reconstruction. Unlike conventional models, such as the KDM, which focus on physiological biomarkers, our approach incorporates explicit morbidity and mortality data, enabling more comprehensive modeling of the aging process. In multivariate analyses, our gap-based model demonstrated that existing models fail to fully account for health status independently of CA.

Our BA – CA gap metric serves as an integrated marker, encompassing both health status and mortality risk, and thus provides a unified summary of an individual’s biological aging trajectory. Multitask training enables the model to embed complex relationships among morbidity, mortality, and aging, producing an interpretable and actionable output. This integration supports the use of the BA – CA gap as a comprehensive biomarker for aging.

The choice of training population is crucial in AI-based health modeling. We found that restricting training to a strictly defined normal population, despite its small sample size, skewed gap values and failed to reflect broader population patterns. Conversely, training on more diverse populations improved discrimination of morbidity status. Although we tested models across different feature sets, those using the full feature set performed best in mortality prediction, even when morbidity prediction performance was similar.

Although mortality prediction is a common task in deep learning research, few previous models for BA estimation have explicitly incorporated both morbidity and mortality as learning objectives [23]. While CA data are readily available, labeled outcomes for health events or direct BA phenotypes are insufficient, especially in longitudinal or community-based cohorts [24]. In this context, unsupervised and self-supervised learning methods are particularly advantageous, as they can extract meaningful biological aging signals despite limited labeled data. These approaches offer both adaptability and scalability, making them well-suited for aging research where outcome labels are often unavailable [25,26].

Our findings suggest that a transformer-based BA – CA gap model incorporating morbidity and mortality data more effectively stratifies health status and predicts mortality risk than existing models. However, further prospective and multicenter studies are needed to establish its generalizability and clinical use. Implementation of such AI-based tools must also address ethical considerations, including data privacy, equitable access, and algorithmic transparency.

### Limitations

This study has several limitations. First, the study design does not allow for causal inference regarding the effects of specific comorbidities on biological aging. Future studies or analyses using causal modeling will be necessary to clarify these relationships. Second, our cohort consisted of individuals who voluntarily underwent routine health checkups at a single tertiary academic institution in Korea. This population may not represent the broader community, introducing selection bias and limiting the generalizability of our findings. External validation in larger, more diverse, and population-based cohorts, including different ethnic, demographic, and socioeconomic backgrounds, will be essential. Third, comorbidities were primarily identified through self-reported questionnaires, which can be subject to recall bias and misclassification. Although we attempted to mitigate this by cross-referencing with medication records and laboratory data, residual inaccuracies may persist. Fourth, while most key features had low missingness, we used mean imputation for missing data to ensure transparency and computational efficiency. This approach, however, may introduce bias and reduce variability, potentially affecting model performance. Our model incorporated a missingness mask to help address this issue, but more robust imputation methods should be explored. Fifth, classifying individuals with missing data on key features as normal may have introduced classification bias, resulting in potential misclassification of participants with unrecognized predisease or disease. This could impact the accuracy of health status stratification and model evaluation. Finally, our analysis relied exclusively on health checkup data. Integrating additional data sources, including genomics, proteomics, or digital health metrics, could further enhance the predictive performance, biological relevance, and clinical applicability of BA estimation models.

### Conclusions

We developed a deep learning model that estimates BA by integrating morbidity and mortality data within a unified framework, using unsupervised and self-supervised learning. This BA – CA gap–based approach serves a sensitive, interpretable biomarker of biological aging and holds promise for advancing personalized health management.

## Supplementary material

10.2196/71592 Multimedia Appendix 1 Supplementary tables presenting feature sets, proportions of missing data, model comparisons, disease-specific analyses, and regression analyses.

10.2196/71592 Checklist 1 STROBE checklist.

## Abbreviations

AI: artificial intelligence
BA: biological age
CA: chronological age
DBP: diastolic blood pressure
DM: diabetes mellitus
DNN: deep neural network
HbA_1c_: glycated hemoglobin
HDL-C: high-density lipoprotein cholesterol
*ICD-10*: *International Classification of Diseases, Tenth Revision*
IRB: institutional review board
KDM: Klemera and Doubal’s method
LDL-C: low-density lipoprotein cholesterol
MI: mutual information
PCC: Pearson correlation coefficient
SBP: systolic blood pressure
SROCC: Spearman rank-order correlation coefficient
STROBE: Strengthening the Reporting of Observational Studies in Epidemiology

## References

[1] Hamczyk MR, Nevado RM, Barettino A, Fuster V, Andrés V (2020). Biological versus chronological aging: JACC focus seminar. J Am Coll Cardiol.

[2] Tian YE, Cropley V, Maier AB, Lautenschlager NT, Breakspear M, Zalesky A (2023). Heterogeneous aging across multiple organ systems and prediction of chronic disease and mortality. Nat Med.

[3] Melzer D, Pilling LC, Ferrucci L (2020). The genetics of human ageing. Nat Rev Genet.

[4] Wen J, Tian YE, Skampardoni I (2024). The genetic architecture of biological age in nine human organ systems. Nat Aging.

[5] Sabayan B, Doyle S, Rost NS, Sorond FA, Lakshminarayan K, Launer LJ (2023). The role of population-level preventive care for brain health in ageing. Lancet Healthy Longev.

[6] Elliott ML, Caspi A, Houts RM (2021). Disparities in the pace of biological aging among midlife adults of the same chronological age have implications for future frailty risk and policy. Nat Aging.

[7] Crane PA, Wilkinson G, Teare H (2022). Healthspan versus lifespan: new medicines to close the gap. Nat Aging.

[8] Rutledge J, Oh H, Wyss-Coray T (2022). Measuring biological age using omics data. Nat Rev Genet.

[9] Ashiqur Rahman S, Giacobbi P, Pyles L, Mullett C, Doretto G, Adjeroh DA (2021). Deep learning for biological age estimation. Brief Bioinform.

[10] Bafei SEC, Shen C (2023). Biomarkers selection and mathematical modeling in biological age estimation. NPJ Aging.

[11] Chua M, Kim D, Choi J (2023). Tackling prediction uncertainty in machine learning for healthcare. Nat Biomed Eng.

[12] Zhavoronkov A, Bischof E, Lee KF (2021). Artificial intelligence in longevity medicine. Nat Aging.

[13] Qiu W, Chen H, Kaeberlein M, Lee SI (2023). ExplaiNAble BioLogical Age (ENABL Age): an artificial intelligence framework for interpretable biological age. Lancet Healthy Longev.

[14] Rahman SA, Adjeroh DA (2020). Centroid of age neighborhoods: a new approach to estimate biological age. IEEE J Biomed Health Inform.

[15] Li X, Ploner A, Wang Y (2020). Longitudinal trajectories, correlations and mortality associations of nine biological ages across 20-years follow-up. Elife.

[16] Kim MK, Han K, Lee SH (2022). Current trends of big data research using the Korean National Health Information Database. Diabetes Metab J.

[17] World Medical Association (2025). World Medical Association Declaration of Helsinki: ethical principles for medical research involving human participants. JAMA.

[18] Elm E von, Altman DG, Egger M, Pocock SJ, Gøtzsche PC, Vandenbroucke JP (2007). Strengthening the Reporting of Observational Studies in Epidemiology (STROBE) statement: guidelines for reporting observational studies. BMJ.

[19] Choromanski KM, Likhosherstov V, Dohan D, Song X, Gane A, Sarlos T Rethinking attention with performers.

[20] Heo B, Chun S, Oh SJ, Han D, Yun S, Kim G AdamP: slowing down the slowdown for momentum optimizers on scale-invariant weights.

[21] Bae CY, Im Y, Lee J (2021). Comparison of biological age prediction models using clinical biomarkers commonly measured in clinical practice settings: AI techniques vs. traditional statistical methods. Front Anal Sci.

[22] Klemera P, Doubal S (2006). A new approach to the concept and computation of biological age. Mech Ageing Dev.

[23] Jiang M, Tian S, Liu S (2024). Accelerated biological aging elevates the risk of cardiometabolic multimorbidity and mortality. Nat Cardiovasc Res.

[24] Rani V, Nabi ST, Kumar M, Mittal A, Kumar K (2023). Self-supervised learning: a succinct review. Arch Comput Methods Eng.

[25] Wang Y, Zhao Y, Therneau TM (2020). Unsupervised machine learning for the discovery of latent disease clusters and patient subgroups using electronic health records. J Biomed Inform.

[26] Azizi S, Culp L, Freyberg J (2023). Robust and data-efficient generalization of self-supervised machine learning for diagnostic imaging. Nat Biomed Eng.

