# Supplementary material for: Biological Age Estimation From the Age Gap Using Deep Learning Integrating Morbidity and Mortality: Model Development and Validation Study
Source: J Med Internet Res. 2025 Sep 10;27:e71592. doi: 10.2196/71592 (PMC12422742; doi:10.2196/71592)
Supplement: Multimedia Appendix 1 [file jmir-v27-e71592-s001.docx]

**Multimedia Appendix 1**

Seong-Eun Moon, Ji Won Yoon, Jae Hyun Bae, Shinyoung Joo, Yoo Hyung Kim, Bon Hyang Lee, Seokho Yoon, Haanju Yoo, Young Min Cho. Biological Age Estimation from the Age Gap: Unsupervised and Self-Supervised Deep Learning Integrating Morbidity and Mortality.

**Table S1.** Base feature set: phenotype domains and associated laboratory and anthropometric measurements.

| **Characteristic** | **Features** |
| --- | --- |
| Anemia | Red blood cell count, mean corpuscular volume |
| Adiposity | Body mass index, waist circumference, skeletal muscle mass, body fat mass, fat-free mass |
| Inflammation | High-sensitivity C-reactive protein |
| Kidney function | Serum creatinine (to calculate estimated glomerular filtration rate) |
| Lung function | Forced expiratory volume in one second, forced vital capacity |
| Metabolism | Glycated hemoglobin |
| Nutrition | Albumin |

**Table S2.** Components of the entire feature set.

| **Category** | **Features** |
| --- | --- |
| Blood tests | Red blood cell count, hemoglobin, hematocrit, red cell distribution width, mean corpuscular volume, mean corpuscular hemoglobin, mean corpuscular hemoglobin concentration, white blood cell count, platelet count, plateletcrit, mean platelet volume, platelet distribution width, erythrocyte sedimentation rate, total protein, albumin, aspartate aminotransferase, alanine transaminase, gamma-glutamyl transferase, alkaline phosphatase, total bilirubin, prothrombin time, activated partial thromboplastin time, lactate dehydrogenase, blood urea nitrogen, serum creatinine, sodium, potassium, chloride, total carbon dioxide content, calcium, phosphorous, uric acid, fasting plasma glucose, glycated hemoglobin, insulin, total cholesterol, triglyceride, high-density lipoprotein cholesterol, low-density lipoprotein cholesterol, apolipoprotein A1, apolipoprotein B, high-sensitivity C-reactive protein, free thyroxine, free triiodothyronine, triiodothyronine, thyroid-stimulating hormone, 25-hydroxyvitamin D, testosterone, free testosterone, estradiol, follicular stimulating hormone, luteinizing hormone, alpha-fetoprotein, carcinoembryonic antigen, cancer antigen 19-9, prostate-specific antigen, cancer antigen 125, Helicobacter pylori IgG antibody |
| Urine tests | Microalbumin, creatinine, microalbumin/creatinine ratio |
| Vital signs | Systolic blood pressure, diastolic blood pressure, pulse rate |
| Anthropometry | Height, weight, body mass index, waist circumference, skeletal muscle mass, body fat mass, fat-free mass, visual acuity |
| Lung function | Forced expiratory volume in one second (FEV1), forced vital capacity (FVC), FEV1/FVC |

**Table S3.** Proportion of missing values for key features.

| **Feature** | **Missing rate (%)** |
| --- | --- |
| Red blood cell count | 2.09 |
| Mean corpuscular volume | 2.09 |
| Body mass index | 2.70 |
| Waist circumference | 2.98 |
| Skeletal muscle mass | 15.19 |
| Body fat mass | 15.19 |
| Fat-free mass | 15.19 |
| High-sensitivity C-reactive protein | 23.43 |
| Serum creatinine | 2.11 |
| Forced expiratory volume in one second | 8.22 |
| Forced vital capacity | 8.22 |
| Glycated hemoglobin | 6.06 |
| Albumin | 2.08 |
| Fasting plasma glucose | 2.09 |
| Triglyceride | 2.35 |
| High-density lipoprotein cholesterol | 2.36 |
| Low-density lipoprotein cholesterol | 38.11 |
| Systolic blood pressure | 2.46 |
| Diastolic blood pressure | 2.46 |

**Table S4.** Comparison of biological and chronological age gaps among models in the normal population.

| **Feature set** | **Morbidity status** | **Men** | | | | **Women** | | | |
| --- | --- | --- | --- | --- | --- | --- | --- | --- | --- |
|  |  | **KDM** | **CAC** | **DNN** | **Proposed** | **KDM** | **CAC** | **DNN** | **Proposed** |
| Base | Normal | 0.85 (−0.95, 2.66) | 0.80 (−0.41, 2.00) | −0.44 (−2.06, 1.18) | 0.59 (0.05, 1.12)^b^ | −0.24 (−0.98, 0.51)^b^ | 1.17 (0.65, 3.94) | −0.10 (−0.52, 0.32) | −0.22 (−0.37, −0.07)^a,b^ |
|  | Pre-disease | −2.20 (−2.32, −2.08) | −1.74 (−1.82, −1.67) | −2.14 (−2.24, −2.04) | 2.18 (2.14, 2.22)^b^ | 8.46 (8.33, 8.58)^b^ | 2.41 (2.33, 2.49) | −3.51 (−3.58, −3.44) | 0.63 (0.60, 0.66)^a,b^ |
|  | Disease | 3.08 (2.98, 3.19) | −5.29 (−5.33, −5.24) | −4.55 (−4.61, −4.48) | 3.90 (3.87, 3.92)^b^ | 19.42 (19.28, 19.57)^b^ | 1.64 (1.58, 1.71) | −8.30 (−8.38, −8.22) | 2.45 (2.41, 2.50)^a,b^ |
| Morbidity-related | Normal | −0.10 (−1.74, 1.54)^b^ | 1.64 (0.50, 2.77) | 0.26 (−1.02, 1.54) | 1.87 (1.04, 2.70)^b^ | −0.79 (−1.50, −0.08)^a,b^ | 1.10 (0.61, 1.59) | −0.33 (−0.75, 0.10) | 2.08 (1.84, 2.33)^b^ |
|  | Pre-disease | 3.91 (3.81, 4.02)^b^ | −1.23 (−1.30, −1.16) | −4.44 (−4.55, −4.33) | 3.35 (3.29, 3.40)^b^ | 12.59 (12.47, 12.71)^a,b^ | 5.97 (5.90, 6.04) | −2.62 (−2.69, −2.55) | 6.06 (6.00, 6.11)^b^ |
|  | Disease | 11.94 (11.85, 12.03)^b^ | −4.82 (−4.87, −4.77) | −14.25 (−14.33, −14.16) | 5.64 (5.61, 5.68)^b^ | 24.86 (24.71, 25.01)^a,b^ | 3.63 (3.57, 3.69) | −9.13 (−9.21, −9.06) | 7.89 (7.83, 7.95)^b^ |
| Entire | Normal | −0.76 (−2.01, 0.49) | −0.96 (−2.10, 0.17) | −0.73 (−1.79, 0.34) | 0.54 (0.11, 0.96) | −0.16 (−0.76, −0.43)^b^ | −0.28 (−0.74, 0.18) | −1.82 (−2.17, −1.47) | −0.05 (−0.08, 0.19)^b^ |
|  | Pre-disease | −0.07 (−0.15, 0.02) | −3.59 (−3.66, −3.52) | 0.17 (0.09, 0.24) | 0.18 (0.16, 0.21) | 6.62 (6.54, 6.71)^b^ | 1.83 (1.77, 1.90) | −3.19 (−3.25, −3.14) | 1.08 (1.06, 1.11)^b^ |
|  | Disease | 1.27 (1.20, 1.34) | −7.35 (−7.41, −7.32) | −3.28 (−3.34, −3.22) | 0.95 (0.93, 0.96) | 10.91 (10.81, 11.01)^b^ | 1.21 (1.14, 1.27) | −6.00 (−6.06, −5.93) | 1.89 (1.87, 1.92)^b^ |

Data are shown as mean gap (95% confidence interval) between biological and chronological ages. ^a^Fulfillment of both negative gap values for normal individuals and positive gap values for individuals with diseases. ^b^Statistically significant differences in the gap values between morbidity statuses.

CAC, chronological age cluster; DNN, deep neural network; KDM, Klemera and Doubal’s method.

**Table S5.** Comparison of biological and chronological age gaps among models in the normal and predisease population.

| **Feature set** | **Morbidity status** | **Men** | | | | **Women** | | | |
| --- | --- | --- | --- | --- | --- | --- | --- | --- | --- |
|  |  | **KDM** | **CAC** | **DNN** | **Proposed** | **KDM** | **CAC** | **DNN** | **Proposed** |
| Base | Normal | 2.37 (0.62, 4.12) | −1.94 (−3.60, −0.14) | 3.73 (2.41, 4.94) | −0.67 (−0.91, −0.22) | −9.70 (−10.83, −8.56)^a,b^ | −4.47 (−4.97, −3.94)^a,b^ | 1.31 (0.93, 1.74) | −0.66 (−0.80, −0.52)^a,b^ |
|  | Pre-disease | 0.03 (−0.27, 0.33) | −0.89 (−1.15, −0.62) | 1.71 (1.51, 1.91) | −0.16 (−0.24, −0.09) | −0.01 (−0.44, 0.42)^a,b^ | −2.24 (−2.45, −2.03)^a,b^ | 0.44 (0.29, 0.58) | 0.19 (0.13, 0.24)^a,b^ |
|  | Disease | 5.06 (4.97, 5.15) | 2.33 (2.26, 2.40) | −0.50 (−0.55, −0.45) | 1.55 (1.52, 1.58) | 14.68 (14.48, 14.89)^a,b^ | 1.53 (1.45, 1.60)^a,b^ | −0.99 (−1.05, −0.93) | 2.77 (2.75, 2.79)^a,b^ |
| Morbidity-related | Normal | −3.93 (−5.45, −2.39)^a,b^ | −9.21 (−10.74, −7.77)^a,b^ | 1.57 (0.50, 2.59) | −0.71 (−1.18, −0.31)^a,b^ | −12.29 (−13.04, −11.54)^a,b^ | −9.80 (−10.26, −9.35)^a,b^ | −0.11 (−0.52, 0.31) | 0.31 (0.19, 0.46) |
|  | Pre-disease | −0.16 (−0.43, 0.11)^a,b^ | −0.08 (−0.34, 0.18)^a,b^ | 0.14 (−0.05, 0.33) | 1.70 (1.58, 1.83)^a,b^ | −0.21 (−0.50, 0.09)^a,b^ | −1.44 (−1.66, −1.22)^a,b^ | 0.60 (0.45, 0.74) | 0.22 (0.17, 0.27) |
|  | Disease | 10.68 (10.58, 10.78)^a,b^ | 6.44 (6.37, 6.50)^a,b^ | −1.38 (−1.43, −1.32) | 6.37 (6.32, 6.42)^a,b^ | 16.24 (16.08, 16.40)^a,b^ | 5.88 (5.79, 5.96)^a,b^ | −0.38 (−0.44, −0.32) | −0.35 (−0.37, −0.33) |
| Entire | Normal | −2.91 (−4.41, −1.35)^a,b^ | −3.60 (−5.78, −1.49)^a,b^ | −0.24 (−1.36, 0.80) | −2.13 (−2.49, −1.71)^a,b^ | −8.96 (−9.61, −8.31)^a,b^ | −6.01 (−6.53, −5.50)^a,b^ | 1.44 (1.10, 1.79) | −1.56 (−1.68, −1.45)^a,b^ |
|  | Pre-disease | 0.04 (−0.18, 0.27)^a,b^ | −0.07 (−0.34, 0.20)^a,b^ | −0.21 (−0.36, −0.05) | −0.06 (−0.12, −0.00)^a,b^ | 0.14 (−0.11, 0.39)^a,b^ | −1.75 (−1.98, −1.53)^a,b^ | 1.20 (1.08, 1.32) | 0.26 (0.21, 0.30)^a,b^ |
|  | Disease | 3.32 (3.25, 3.39)^a,b^ | 2.75 (2.69, 2.82)^a,b^ | −1.09 (−1.13, −1.05) | 2.11 (2.10, 2.13)^a,b^ | 11.89 (11.77, 12.02)^a,b^ | 4.83 (4.74, 4.92)^a,b^ | 0.34 (0.29, 0.39) | 2.28 (2.26, 2.30)^a,b^ |

Data are shown as mean gap (95% confidence interval) between biological and chronological ages. ^a^Fulfillment of both negative gap values for normal individuals and positive gap values for individuals with diseases. ^b^Statistically significant differences in the gap values between morbidity statuses.

CAC, chronological age cluster; DNN, deep neural network; KDM, Klemera and Doubal’s method.

**Table S6.** Comparison of biological and chronological age gaps among models in entire population excluding outliers.

| **Feature set** | **Morbidity status** | **Men** | | | | **Women** | | | |
| --- | --- | --- | --- | --- | --- | --- | --- | --- | --- |
|  |  | **KDM** | **CAC** | **DNN** | **Proposed** | **KDM** | **CAC** | **DNN** | **Proposed** |
| Base | Normal | 4.03 (2.88, 5.19) | −6.58 (−7.58, −5.57)^a,b^ | −1.10 (−1.87, −0.34)^a,b^ | −2.83 (−3.00, −2.66)^a,b^ | −6.88 (−7.67, −6.09)^a,b^ | −4.93 (−5.33, −4.52)^a,b^ | 3.27 (2.97, 3.54) | −1.61 (−1.70, −1.52)^a,b^ |
|  | Pre-disease | 2.94 (2.73, 7.48) | −3.55 (−3.73, −3.36)^a,b^ | 1.33 (1.20, 1.46)^a,b^ | −1.11 (−1.15, −1.08)^a,b^ | 3.61 (3.35, 3.88)^a,b^ | −0.70 (−0.85, −0.55)^a,b^ | 3.75 (3.65, 3.84) | 0.44 (0.40, 0.48)^a,b^ |
|  | Disease | 7.48 (7.37, 7.60) | 0.72 (0.64, 0.81) | 1.69 (1.63, 1.75)^a,b^ | 0.46 (0.44, 0.48)^a,b^ | 11.36 (11.16, 11.56)^a,b^ | 3.15 (3.07, 3.24)^a,b^ | 2.99 (2.93, 3.05) | 3.78 (3.74, 3.82)^a,b^ |
| Morbidity-related | Normal | −5.42 (−6.52, 4.30)^a,b^ | −12.24 (−13.09, 11.40)^a,b^ | 1.67 (0.96, 2.41) | −5.55 (−5.61, −5.48)^a,b^ | −12.13 (−12.69, −11.58)^a,b^ | −9.14 (−9.46, −8.82)^a,b^ | −0.28 (−0.61, 0.03)^b^ | −4.64 (−4.68, −4.61)^a,b^ |
|  | Pre-disease | −1.27 (−1.48, −1.06)^a,b^ | −5.41 (−5.59, −5.23)^a,b^ | 2.16 (2.04, 2.29) | −2.66 (−2.69, −2.63)^a,b^ | 0.78 (0.59, 0.98)^a,b^ | −1.93 (−2.07, −1.79)^a,b^ | 2.02 (1.92, 2.12)^b^ | −0.84 (−0.86, −0.81)^a,b^ |
|  | Disease | 11.62 (11.48, 11.77)^a,b^ | 2.48 (2.40, 2.55)^a,b^ | 2.48 (2.42, 2.53) | 0.27 (0.25, 0.28)^a,b^ | 12.58 (12.41, 12.75)^a,b^ | 4.94 (4.85, 5.03)^a,b^ | 3.47 (3.40, 3.53)^†^ | 1.85 (1.83, 1.87)^a,b^ |
| Entire | Normal | 1.32 (0.26, 2.40) | −8.27 (−9.31, −7.23)^a,b^ | −1.03 (−1.64, −0.45) | −4.80 (−4.88, −4.72)^a,b^ | −2.24 (−2.36, −2.12)^a,b^ | −6.49 (−6.84, −6.13)^a,b^ | 0.79 (0.53, 1.04)^b^ | −4.11 (−4.16, −4.06)^a,b^ |
|  | Pre-disease | 1.21 (1.02, 1.40) | −1.59 (−1.78, −1.40)^a,b^ | 0.21 (0.10, 0.31) | −2.14 (−2.18, −2.11)^a,b^ | 0.34 (0.30, 0.38)^a,b^ | −1.91 (−2.05, −1.77)^a,b^ | 2.14 (2.05, 2.22)^b^ | −1.04 (−1.07, −1.01)^a,b^ |
|  | Disease | 5.84 (5.73, 5.96) | 0.98 (0.91, 1.06)^a,b^ | 0.20 (0.14, 0.26) | 1.18 (1.16, 1.20)^a,b^ | 2.57 (2.54, 2.60)^a,b^ | 3.70 (3.60, 3.79)^a,b^ | 3.35 (3.29, 3.41)^b^ | 1.20 (1.18, 1.22)^a,b^ |

Data are shown as mean gap (95% confidence interval) between biological and chronological ages. ^a^Fulfillment of both negative gap values for normal individuals and positive gap values for individuals with diseases. ^b^Statistically significant differences in the gap values between morbidity statuses.

CAC, chronological age cluster; DNN, deep neural network; KDM, Klemera and Doubal’s method.

**Table S7.** Comparison of biological and chronological age gaps among models in the entire population.

| **Feature set** | **Morbidity status** | **Men** | | | | **Women** | | | |
| --- | --- | --- | --- | --- | --- | --- | --- | --- | --- |
|  |  | **KDM** | **CAC** | **DNN** | **Proposed** | **KDM** | **CAC** | **DNN** | **Proposed** |
| Base | Normal | 5.59 (2.99, 7.84) | −0.04 (−2.01, 1.47) | 3.31 (2.09, 4.37) | −2.23 (−2.48, −1.94)^a,b^ | −22.25 (−24.95, −20.05)^a,b^ | −3.14 (−3.77, −2.54) | 1.76 (1.35, 2.18) | −0.59 (−0.71, −0.49)^a,b^ |
|  | Pre-disease | −2.49 (−2.93, −2.02) | −2.85 (−3.15, −2.57) | 1.08 (0.90, 1.29) | −0.75 (−0.81, −0.70)^a,b^ | −10.43 (−11.33, −9.54)^a,b^ | −3.58 (−3.82, −3.34) | 0.49 (0.34, 0.64) | −0.31 (−0.35, −0.26)^a,b^ |
|  | Disease | 0.80 (0.50, 1.09) | −1.66 (−1.85, −1.48) | −0.57 (−0.69, −0.45) | 0.49 (0.45, 0.53)^a,b^ | 9.50 (8.57, 10.44)^a,b^ | −1.28 (−1.51, −1.04) | −1.06 (−1.21, −0.91) | 0.66 (0.61, 0.71)^a,b^ |
| Morbidity-related | Normal | −0.80 (−3.34, 1.53) | −10.74 (−13.02, −8.92)^b^ | 1.19 (−0.13, 2.27) | −4.21 (−4.33, −4.13)^a,b^ | −22.68 (−24.94, −18.95)^a,b^ | −7.78 (−8.26, −7.29)^b^ | 0.52 (0.13, 0.91) | −3.11 (−3.17, −3.06)^a,b^ |
|  | Pre-disease | −5.55 (−5.99, −5.12) | −5.39 (−5.68, −5.10)^b^ | 0.56 (0.39, 0.77) | −1.54 (−1.57, −1.50)^a,b^ | −10.00 (−10.71, −8.84)^a,b^ | −5.04 (−5.25, −4.83)^b^ | 0.57 (0.42, 0.71) | −1.11 (−1.15, −1.08)^a,b^ |
|  | Disease | 2.35 (2.05, 2.66) | 0.12 (−0.05, 0.28)^b^ | −0.10 (−0.22, 0.22) | 0.29 (0.26, 0.33)^a,b^ | 8.51 (7.61, 9.77)^a,b^ | 0.19 (−0.01, 0.40)^b^ | −0.02 (−0.15, 0.13) | 0.69 (0.64, 0.73)^a,b^ |
| Entire | Normal | 6.06 (2.03, 10.06) | −1.62 (−3.49, 0.29) | 0.02 (−0.87, 0.89) | −3.73 (−3.89, −3.49)^a,b^ | −1.62 (−1.69, −1.50)^a,b^ | −4.15 (−4.75, −3.56) | 0.31 (−0.00, 0.63) | −3.29 (−3.37, −3.23)^a,b^ |
|  | Pre-disease | −0.37 (−1.01, 0.26) | −2.06 (−2.42, −1.73) | 0.60 (0.45, 0.74) | −0.81 (−0.85, −0.76)^a,b^ | −0.70 (−0.73, −0.66)^a,b^ | −4.79 (−5.03, −4.54) | 0.40 (0.29, 0.51) | −0.99 (−1.02, −0.94)^a,b^ |
|  | Disease | 0.19 (−0.30, 0.67) | −3.45 (−3.68, −3.22) | 0.19 (0.10, 0.29) | 0.47 (0.43, 0.51)^a,b^ | 0.63 (0.59, 0.67)^a,b^ | −4.30 (−4.58, −4.04) | −0.37 (−0.48, −0.26) | 1.19 (1.14, 1.23)^a,b^ |

Data are shown as mean gap (95% confidence interval) between biological and chronological ages. ^a^Fulfillment of both negative gap values for normal individuals and positive gap values for individuals with diseases. ^b^Statistically significant differences in the gap values between morbidity statuses.

CAC, chronological age cluster; DNN, deep neural network; KDM, Klemera and Doubal’s method.

**Table S8.** Gaps between biological and chronological ages by glycemic status.

| **Population** | **Normal** | **Prediabetes** | **Diabetes mellitus** |
| --- | --- | --- | --- |
| Men | −2.23 (−2.48, −1.94)^a,b^ | 0.39 (0.30, 0.47)^a,b^ | 4.35 (4.27, 4.48)^a,b^ |
| Women | −0.59 (−0.71, −0.49)^a,b^ | 0.83 (0.75, 0.91)^a,b^ | 3.29 (3.17, 3.42)^a,b^ |

Data are shown as mean gap (95% confidence interval) between biological and chronological ages. Model trained on the entire population with the entire feature set. ^a^Fulfillment of both negative gap values for normal individuals and positive gap values for individuals with diseases. ^b^Statistically significant differences in the gap values between morbidity statuses.

**Table S9.** Gaps between biological and chronological ages by blood pressure status.

| **Population** | **Normal** | **Elevated blood pressure** | **Hypertension** |
| --- | --- | --- | --- |
| Men | −2.23 (−2.48, −1.94)^a,b^ | −0.65 (−0.74, −0.57)^a,b^ | 0.69 (0.61, 0.75)^a,b^ |
| Women | −0.59 (−0.71, −0.49) | −0.72 (−0.82, −0.62) | 0.27 (0.17, 0.36) |

Data are shown as mean gap (95% confidence interval) between biological and chronological ages. Model trained on the entire population with the entire feature set. ^a^Fulfillment of both negative gap values for normal individuals and positive gap values for individuals with diseases. ^b^Statistically significant differences in the gap values between morbidity statuses.

**Table S10.** Gaps between biological and chronological ages by lipid profiles.

| **Population** | **Normal** | **Borderline lipid levels** | **Dyslipidemia** |
| --- | --- | --- | --- |
| Men | −2.23 (−2.48, −1.94)^a,b^ | −0.71 (−0.77, −0.65)^a,b^ | 0.80 (0.75, 0.86)^a,b^ |
| Women | −0.59 (−0.71, −0.49)^a,b^ | −0.35 (−0.40, −0.31)^a,b^ | 0.13 (0.05, 0.23)^a,b^ |

Data are shown as mean gap (95% confidence interval) between biological and chronological ages. Model trained on the entire population with the entire feature set. ^a^Fulfillment of both negative gap values for normal individuals and positive gap values for individuals with diseases. ^b^Statistically significant differences in the gap values between morbidity statuses.

**Table S11.** Gaps between biological and chronological ages by cardiovascular disease status.

| **Population** | **Normal** | **Cardiovascular disease** |
| --- | --- | --- |
| Men | −2.23 (−2.48, −1.94)^a,b^ | 1.19 (0.99, 1.46)^a,b^ |
| Women | −0.59 (−0.71, −0.49)^a,b^ | 0.57 (0.23, 0.94)^a,b^ |

Data are shown as mean gap (95% confidence interval) between biological and chronological ages. Model trained on the entire population with the entire feature set. ^a^Fulfillment of both negative gap values for normal individuals and positive gap values for individuals with diseases. ^b^Statistically significant differences in the gap values between morbidity statuses.

**Table S12.** Gaps between biological and chronological ages by cancer status.

| **Population** | **Normal** | **Cancer** |
| --- | --- | --- |
| Men | −2.23 (−2.48, −1.94)^a,b^ | 0.56 (0.25, 0.85)^a,b^ |
| Women | −0.59 (−0.71, −0.49)^b^ | −0.08 (−0.27, −0.12)^b^ |

Data are shown as mean gap (95% confidence interval) between biological and chronological ages. Model trained on the entire population with the entire feature set. ^a^Fulfillment of both negative gap values for normal individuals and positive gap values for individuals with diseases. ^b^Statistically significant differences in the gap values between morbidity statuses.

**Table S13.** Linear regression analyses for time-to-death using chronological and biological ages in the entire population.

| **Population** | **Model** | **Slope** | | ***R^2^*** | | **Pearson correlation coefficient** | |
| --- | --- | --- | --- | --- | --- | --- | --- |
|  |  | **CA** | **BA** | **CA** | **BA** | **CA** | **BA** |
| Men | KDM using BA | −4.683 | −8.255^a^ | 0.001 | 0.051 | −0.032 | −0.227 |
|  | CAC using BA | −4.683 | −13.487^a^ | 0.001 | 0.030 | −0.032 | −0.172 |
|  | DNN using BA | −4.683 | −3.732 | 0.001 | 0.001 | −0.032 | −0.024 |
|  | Gap-based using BA | −4.683 | −19.938^a^ | 0.001 | 0.020 | −0.032 | −0.141 |
|  | KDM using CA and BA–CA gap | −4.683 | −8.753^a^ | 0.001 | 0.051 | −0.032 | −0.227 |
|  | CAC using CA and BA–CA gap | −4.683 | −14.891^a^ | 0.001 | 0.031 | −0.032 | −0.168 |
|  | DNN using CA and BA–CA gap | −4.683 | −0.602 | 0.001 | 0.001 | −0.032 | 0.013 |
|  | Gap-based using CA and BA–CA gap | −4.683 | −153.599^a^ | 0.001 | 0.120 | −0.032 | −0.342 |
| Women | KDM using BA | 3.794 | 4.537 | 0.001 | 0.001 | 0.029 | 0.034 |
|  | CAC using BA | 3.794 | −3.779 | 0.001 | 0.002 | 0.029 | −0.046 |
|  | DNN using BA | 3.794 | 2.654 | 0.001 | 0.000 | 0.029 | 0.017 |
|  | Gap-based using BA | 3.794 | −3.823 | 0.001 | 0.001 | 0.029 | −0.032 |
|  | KDM using CA and BA–CA gap | 3.794 | 23.594 | 0.001 | 0.002 | 0.029 | 0.026 |
|  | CAC using CA and BA–CA gap | 3.794 | −6.252 | 0.001 | 0.005 | 0.029 | −0.072 |
|  | DNN using CA and BA–CA gap | 3.794 | −14.627 | 0.001 | 0.001 | 0.029 | −0.021 |
|  | Gap-based using CA and BA–CA gap | 3.794 | −167.651^a^ | 0.001 | 0.086 | 0.029 | −0.274 |

^a^*P* < 0.001. BA, biological age; CA, chronological age; CAC, chronological age cluster; DNN, deep neural network; KDM, Klemera and Doubal’s method.
